# Yeast-fermented feed improves growth and meat quality in meat duck: Integrated metabolomics and proteomics reveal amino acid metabolic mechanisms

**DOI:** 10.1016/j.psj.2026.107366

**Published:** 2026-06-30

**Authors:** Yang Liu, Hailin Liu, Ping Deng, Chuang Li, Xuan Huang, Xu Zhang, Xiaolong Peng, Zhicai Li, Yan Hu, Chao Peng, Qiuzhong Dai

**Affiliations:** Institute of Animal Husbandry and Veterinary Medicine, Hunan Academy of Agricultural Sciences, Changsha 410131, Hunan, China

**Keywords:** Fermented feed, Duck, Meat quality, Metabolomics, Proteomics

## Abstract

Fermented feed is increasingly used in monogastric animal production to support growth, but little is known about its effects on meat quality or the mechanisms drive these effects. We fed 240 one-day-old Cherry Valley ducks either a basal diet or the same diet supplemented with 5% yeast-fermented feed for 42 days. Growth performance and carcass traits were recorded at slaughter, Breast muscle was analyzed for quality, chemical properties, amino acid composition, and subjected to metabolomics and proteomics. Results showed that ducks fed yeast-fermented feed had higher body weight at 42-d-old (*p =* 0.042), higher body weight gain from 15-d-old to 42-d-old (*p =* 0.036), and lower feed to gain ratio from 15-d-old to 42-d-old (*p =* 0.032) and the whole experiment period (*p =* 0.046) compared with the control group. Additionally, ducks fed yeast-fermented feed had higher semi-eviscerated yield (*p =* 0.001) and eviscerated yield (*p =* 0.001) compared with control group. Moreover, breast muscle from ducks fed yeast-fermented feed contained less crude fat (*p =* 0.023) but more phenylalanine (*p =* 0.004) and lysine (*p =* 0.038) than the control group. Omics analysis identified 179 differentially abundant metabolites and 388 differentially expressed proteins, converging on three amino acid-related pathways including glycine, serine and threonine metabolism, arginine and proline metabolism, and aminoacyl-tRNA biosynthesis. Notably, glycocyamine, sarcosine, creatine, creatinine, glycine amidinotransferase (GATM), guanidinoacetate N-methyltransferase (GAMT), and creatine kinase U-type, mitochondrial (Ckmt1) were up-regulated in duck breast muscle by dietary supplement of yeast-fermented feed. Correlation analysis revealed 24 significant associations among key molecules, and multiple metabolites and proteins correlated with meat quality traits including crude fat content, phenylalanine, and lysine. In conclusion, dietary supplement of 5% yeast-fermented feed improves meat duck growth performance during the late phase and decreased feed to gain ratio for the whole experimental period, enhanced carcass yields and reduced breast muscle crude fat while increasing phenylalanine and lysine. Multi-omics integration revealed glycine, serine and threonine metabolism and arginine and proline metabolism as central regulatory pathways, with creatine biosynthesis enzymes and metabolites identified as key biomarkers. These findings provide a mechanistic basis for using yeast-fermented feed to enhance duck meat quality.

## Introduction

Duck meat provides high-quality protein, essential vitamins, and polyunsaturated fatty acids, positioning it as a valuable component of human diets (Tang et al., 2023). However, the meat duck industry faces a dual challenge, that production must expand to meet rising consumer demand for duck meat, yet modern intensive farming systems and artificial breeding have prioritized rapid growth and yield at the expense of meat quality (Huo et al., 2021; Li et al., 2024). This trade-off has created a pressing need for nutritional strategies that can restore quality attributes while maintaining production efficiency. Meat quality itself operates on two levels (Zhang et al., 2021). Appearance quality, including traits such as meat color and texture, shape consumer decisions at the point of purchase. These visual cues are immediately accessible and strongly influence purchasing decisions. Nutritional quality, encompassing amino acid (**AA**) and fatty acid (**FA**) composition, operates less visibly but is increasingly recognized for its direct links to human health. Though these parameters are more complex to measure and communicate, they are gaining attention from both researchers and health-conscious consumers. Therefore, developing practical nutritional interventions that enhance both dimensions of meat quality has become a priority in modern meat duck production system.

Fermented feed (**FF**) represents one solution for meat quality intervention. The macromolecules and anti-nutritional factors in feed ingredients are converted into more bioavailable and less toxic nutrients through the metabolic activities of microorganisms. The fermentation process simultaneously enhances nutritional value and generates bioactive compounds including organic acids, enzymes, and small peptides. These modifications not only improve feed palatability and digestibility, but also contribute to beneficial effects on gut microbiota and overall animal health (Yang et al., 2021). FFs has been widely adopted in monogastric animal production as an alternative to antibiotic growth promoters and as means to reduce feed cost, with consistent reports of growth performance benefits across multiple species (Shuai et al., 2023; Zhu et al., 2023). However, evidence regarding FF’s effects on meat quality remains scare. A recent study in growing pigs reported that dietary supplementation with 10% FF altered the profile of volatile compounds in pork, enhancing both flavor and umami taste (Liu et al., 2023). This finding gives a hint that FF-mediated benefits may extend beyond growth performance into sensory and nutritional attributes of meat, yet direct evidence in poultry is lacking, and the mechanisms through which FF might modulate meat quality remain unexplored.

Our previous study with Cherry Valley duck (5 experimental groups, 8 replicates per group, and 10 ducks per replicate) showed that dietary supplement of 5% yeast-derived FF was optimal for the growth performance of ducks comparing to the ones with 0, 10%, 15%, and 20% FF (Supplement Table 1). Based on this result, the present study was designed to comprehensively evaluate the effects of dietary supplement of 5% FF on slaughter performance and meat quality in Cherry Vally duck. Moreover, metabolomic and proteomics were integrated to uncover the mechanism underlying observed phenotypic changes. By connecting feeding strategy with meat quality alterations, this study aims to provide valuable insights into the development of sustainable and efficient feeding strategies for poultry industry.

## Materials and methods

The experimental protocols and animal care procedures were approved by the Institutional Animal Care and Use Committee of Hunan Institute of Animal Husbandry and Veterinary Medicine (AHVM20240501). The study followed the ARRIVE 2.0 guidelines for reporting animal experiments (Percie Du Sert et al., 2020).

### Fermented feed preparation

The FF used in the current study was kindly provided by Hubei LucNova Biotechnology Co., Ltd (Huanggang, Hubei, China). The fermentation substrate consisted of white distiller’s grains (**DDGS**) derived from corn-based ethanol production, obtained as a wet by-product flowing alcohol distillation. The raw material was collected within 24 h of distillation, with 88.3% dry matter (**DM**) and 0.48 g/cm^3^ bulk density. The nutrient composition and content of the raw material were 26.5% crude protein (**CP**), 5.9% ether extract (**EE**), 6.6% crude fiber (**CF**), and 4.8% ash on a dry matter basis.

A mixed inoculum containing *Saccharomyces cerevisiae* (NCBI Accession No. MN038413), *Phaffia rhodozyma* (CCTCC M20211124), and *Rhodotorula mucilaginosa* (CCTCC M2022812) was prepared at 10^7^ CFU/g each. Fermentation was started with liquid activation of each yeast strain in yeast peptone dextrose (**YPD**) broth at 30 °C for 12 h, and was continued with solid-state fermentation of the inoculated substrate at 40% moisture and 30 °C for 24 h with continuous aeration. The third state of fermentation was anaerobic maturation in sealed valve-equipped plastic bags at ambient temperature for 48 h. After fermentation, the product was dried at 65 °C to a final moisture content of 10% and stored at 4 °C until use.

Contents of DM, CP, EE, ash, CF, calcium, total phosphorus, and AAs were determined following the procedures of AOAC (2023). Metabolic energy (**ME**) of the finished FF was determined using total excreta collection method with adult male ducks (Schiavone et al., 2017). ME was calculated as: ME = (feed intake × diet gross energy – excreta output × excreta gross energy) / feed intake. Gross energy of both feed and excreta was measured using an adiabatic bomb calorimeter (PARR 6100, Moline, IL). The final nutrient composition of the FF is listed in Table 1.

**Table 1:** Nutrient composition and content of fermented feed (dry matter basis, %).

Item	Content
Metabolic energy, MJ/kg	11.04
Dry matter	90.50
Crude protein	18.50
Ether extract	3.51
Ash	6.84
Crude fiber	4.66
Calcium	0.29
Total phosphorus	0.80
Lysine	1.05
Methionine	0.25
Methionine + Cystine	0.37
Threonine	0.69
Tryptophan	0.15
Arginine	1.18
Histidine	0.48

### Animals and treatments

A total of 240 healthy 1-day-old Cherry Valley ducks of similar body weight were obtained from EcoLovo Co., Ltd (Zhaoqing, China) and randomly allocated to two dietary treatments: a control group (**CON**) receiving basal diet and an FF-treated group (**FFD**) receiving basal diet supplemented with 5% FF. Each treatment comprised 120 ducks, evenly allocated into 12 cages treating as experimental replicates, with 10 ducks per replicate. Length, width, and height of the cage were 1.8 m, 1.2 m, and 2.0 m. Each cage was equipped with a feed bucket and three nipple drinkers on the side. Birds had free access to feed and water, and artificial illumination and regular sanitation were applied throughout the trial. Indoor temperature was maintained at 30 to 35 °C for the first week, and decreased 3 °C each week until reaching 20 °C. The feeding trial lasted 42 days, dived into an early phase (day 1 to 14) and a late phase (day 15 to 42). The ingredient composition and nutrient levels of the experimental diets are presented in Table 2.

**Table 2:** Composition and nutrient contents of diets for CON and FFD (dry matter basis, %).

Items	Early stage (1ཞ14 d)		Late stage (15ཞ42 d)	
	CON	FFD	CON	FFD
Ingredients				
Corns	34.81	34.92	38.86	37.85
Wheat	15.00	15.00	15.00	15.00
Flour	5.00	5.00	5.00	5.00
Rice bran	9.00	6.00	8.00	6.00
Soybean meal	21.60	21.50	14.80	14.70
Corn gluten meal (CP 60%)	3.00	3.00	4.00	4.00
Distillers dried grains with soluble	7.00	5.00	8.00	6.00
Fermented feed	0.00	5.00	0.00	5.00
CaHPO_4_	1.20	1.20	1.00	1.00
Limestone	1.50	1.50	1.50	1.50
NaCl	0.30	0.30	0.30	0.30
Soybean oil	0.00	0.00	2.00	2.15
99% DL-Methionine	0.17	0.18	0.14	0.14
78% l-Lysine H_2_SO_4_	0.42	0.40	0.40	0.36
Premix^1^	1.00	1.00	1.00	1.00
Total	100.00	100.00	100.00	100.00
Nutrient contents^2^				
Metabolic energy, MJ/kg	11.85	11.85	12.56	12.56
Crude protein	20.04	20.03	18.07	18.07
Ether extract	3.82	3.26	5.80	5.50
Calcium	0.91	0.92	0.85	0.86
Total phosphorus	0.67	0.64	0.59	0.58
Available phosphorus	0.36	0.35	0.32	0.32
NaCl	0.51	0.50	0.50	0.49
Lysine	1.25	1.25	1.05	1.04
Methionine	0.50	0.50	0.45	0.45
Methionine + Cystine	0.86	0.85	0.78	0.77
Threonine	0.72	0.73	0.63	0.64
Tryptophan	0.22	0.22	0.18	0.18
Arginine	1.08	1.11	0.88	0.90
Histidine	0.51	0.51	0.45	0.45

1 The premix provides the following per kg of diet: VA 6 500 IU, VD_3_ 2250 IU, VE20 IU, VK_3_ 2.0 mg, VB_1_ 4.0 mg, VB_2_ 3.6 mg, VB_6_ 4.0 mg, VB_12_ 0.02 mg, biotin 0.15 mg, nicotinic acid 15 mg, pantothenate calcium 11 mg, folic acid 1.0 mg, antioxidant 100 mg, Fe 60 mg, Cu 10 mg, Mn 90 mg, Zn 85 mg, I 0.40 mg, Se 0.30 mg.

2 The metabolic energy is calculated value, while the other nutrient contents are measured values.

### Growth performance and carcass traits

On day 1, 15, and 42, the body weight (**BW**) of ducks in each replicate was measured after 12-h fast. Daily feed intake for each replicate was recorded to calculate average daily weight gain (**ADG**), average daily feed intake (**ADFI**), and feed to gain ratio (**F/G**). At the end of trial, 1 ducks per replicate (n = 12/group) were randomly selected for carcass evaluation, following Li et al. (2024). Briefly, selected birds were euthanized by electrical stunning followed by exsanguination. After removal of feathers, beak shells, and foot webs, the carcasses were weighed. Breast and thigh muscle were dissected and weighed, along with abdominal fat and sebum. Semi-eviscerated and eviscerated yields were calculated relative to live weight, and yields of breast muscle, thigh muscle, abdominal fat, and sebum were calculated relative to eviscerated weight.

### Sample collection and meat quality

The left *Pectoralis major* was excised for meat quality evaluation, and the subcutaneous fat and fascia were removed. Meat color was measured at three random location 45 min and 24 h post mortem using a colorimeter (CR-400, Minolta Camera Co., Osaka, Japan), represented by brightness (L*), redness (a*), and yellowness (b*) values. pH value was determined 45 min (**pH_45min_**) and 24 h post-mortem (**pH_24h_**) using a portable pH meter (206-pH3, Testo AG, Titisee-Neustadt, Germany), with the average of three readings recorded. Cooking loss and shear force were evaluated as described by He et al. (2018). Briefly, samples were trimmed to 1 cm × 3 cm × 3 cm chops, weighed, and cooked in an 80 °C water bath until an internal temperature of 75°C was reached. After cooling to room temperature and blotting with absorbent paper, samples were reweighed. Cooking loss was calculated as [(initial weight – cooked weight) / initial weight] × 100. Shear force was measured perpendicular to the muscle fibers direction using a tender meter (C-LM3, Kenuode Technology Co., Chongqing, China). All measurements were performed in triplicate for each sample.

### Meat chemical composition

Moisture, crude protein, and crude fat contents were analyzed following Muñoz-Lapeira et al. (2025). Moisture was determined by drying at 103°C to constant weight. Crude protein was quantified using Kjeldahl nitrogen analyzer (Gerhardt VAPODEST 200, Bonn, Germany) using Kjeldahl method. Crude fat content was measured via acid hydrolysis followed by Soxhlet petroleum ether extraction. Approximately 20 g of minced sample was weighed (**m_0_**), mixed with 120 mL of 2 mol/L HCl, and hydrolyzed in an 80 °C water bath for 1 h. After filtration, the residue was dried at 103°C and weighed (**m_1_**). The dried residue was then extracted with petroleum ether to constant weight (**m_2_**). Crude fat content was calculated as [(m_1_ – m_2_) / m_0_] × 100.

### Meat amino acid profile

AA composition was analyzed as described by Chen et al. (2023). Lyophilized samples (0.1 g) were ground to powders, mixed with 10 mL of HCl (6 mol/L), and hydrolyzed at 110°C for 24 h. After cooling, the hydrolysate was vortexed, diluted with ultrapure water, and filtered through a 0.22 μm membrane. AA were separated using an automatic AA analyzer (L-8900, Hitachi, Tokyo, Japan) equipped with a 4.6 mm × 60 mm ion-exchange column (Hitachi, Tokyo, Japan). The mobile phase consisted of pH-1, −2, −3, −4, and buffer solutions (FUJIFILM Wako, Tokyo Japan) at a flow rate of 0.4 mL/min. The injection volume was 20 μL. Column and reaction unit temperatures were maintained at 57 °C and 130 °C, respectively. Detection was performed at 440 and 570 nm.

### Meat metabolomic analysis

One sample from each replicate was selected for the omics analysis. Metabolite extraction and LC-MS/MS analysis were performed as previously established (Liu et al., 2024). Sample tissues (25 mg) were transferred to 1.5 mL microcentrifuge tubes, mixed with 500 μL of extraction solvent (methanol: acetonitrile: water, 2: 2: 1, v/v/v), and sonicated on ice for 1 h. After precipitation at −20°C for 1 h, samples were centrifuged (14000 × g, 4°C, 20 min). The resulting supernatant was vacuum-dried and reconstituted for liquid chromatograph (**LC-MS**)/mass spectrometer (**MS**) analysis. A pooled quality control (**QC**) sample, prepared by combining equal aliquots from all individual samples, was used for system conditioning and quality assessment.

Chromatographic separation was achieved on a 1290 Infinity LC UPLC system (Agilent Technologies, Santa Clara, USA) equipped with an ACQUITY UPLC BEH Amide column (1.7 μm, Waters, Wexford, Ireland). The mobile phase consisted of (A) water with 0.1% formic acid and (B) acetonitrile with 0.1% formic acid. A linear gradient was applied: 0-2 min, 95% B; 2-12 min, 95-50% B; 12-15 min, 50% B; 15-16 min, 50-95% B; 16-20 min, 95% B. The column temperature was maintained at 40°C with a flow rate of 0.5 mL/min, and samples were held at 4°C during analysis. The injection volume was 2 μL.

Raw MS data were converted using ProteoWizard MSConvert and processed with XCMS for peak detection, retention time alignment, and feature extraction. Metabolite annotation relied on accurate mass measurements (mass error < 25 ppm) and MS/MS spectral matching against Human Metabolome Database (**HMDB**). Features were retained if present in ≥ 50% of samples within at least one experimental group.

### Meat proteomic analysis

Protein extraction and tandem mass tag (**TMT**)-based quantitative proteomic were performed as described by Chen et al. (2024). Samples (0.5 g) were snap-frozen in liquid nitrogen, ground to powders, and lysed in 2 mL of buffer containing 4% sodium dodecyl sulfate, 100 mM dithiothreitol (**DTT**), and 150 mM Tris-HCL (pH 8.5). After homogenization and boiling (5 min), lysates were sonicated and centrifuged (16000 × g, 4 °C, 15 min). Proteins in the supernatant (1 mg) were reduced with 5 mM 1, 4-dithiothreitol (**DTT**, 56 °C, 30 min), alkylated with 11 mM iodoacetamide (room temperature, 20 min, in darkness), and digested with trypsin (protein: trypsin = 50: 1) in 25 mM triethylamine borane at 37 °C for 16 h. Peptides were labeled with TMT reagents (dissolved in 41 μL anhydrous acetonitrile) for 60 min at room temperature, quenched with 5% hydroxylamine (15 min), and lyophilized.

Labeled peptides were fractionated using a ultra-high performance liquid chromatography (**UPLC**) 3000 system (Thermo Fisher, Waltham, MA) equipped with an XBridge BEH 300 C18 column (Waters, Milford, CT). Mobile phase A (water mixed with 0.1% formic acid) and B (acetonitrile mixed with 0.1% formic acid, pH 10) were applied at 0.8 mL/min with a linear gradient: 0-2 min, 2-5% B; 2-42 min, 5-20% B; 42-50 min, 20-35% B; 50-52 min, 35-90% B; 52-60 min, 90% B. Thirty fractions were collected and concatenated to 15 final fractions, which were resuspended in 0.1% formic acid.

Peptides were analyzed on an Easy nLC-1000 system coupled to the Q Exactive mass spectrometer (Thermo Fisher, Waltham, MA). MS1 scans were acquired at 70000 resolution (200 *m/z*) with an automatic gain control (**AGC**) target of 1 × 10^6^ and maximum injection time of 50 ms. MS2 scans (top 15 data-dependent acquisition) used an AGC of 1 × 10^5^, injection time of 100 ms, and high energy collision dissociation (**HCD**) with collision energy of 30 eV. Dynamic exclusion was set to 30 s. Raw data were processed in Proteome Discoverer 2.4 against the UniProt_Antidae.fasta database. Search parameters were trypsin/P digestion, maximum 2 missed cleavages, 20 ppm mass tolerance. Fixed modifications were carbamidomethylation (C), TMT6plex (K), and TMT6Plex (N-term). Peptides (≥ 6 AAs) and proteins (≥ 1 unique peptide) were filtered at 1% false discovery rate (**FDR**). TMT quantification was based on reporter ion intensity. Differentially abundant proteins (**DAPs**) were identified using fold-change threshold of FC > 1.50 or < 0.67 with Student’s t-test *p* < 0.05. Gene ontology (**GO**) annotation was performed using Blast2GO (http://www.balst2go.org), and Kyoto Encyclopedia of Genes and Genomes (**KEGG**) pathways analysis was conducted with significance set up at *p* < 0.05.

### Statistical analysis

The data were analyzed by Student’s *t*-test in Statistical Package for Social Sciences 19.0 (IBM, Armonk, NY). All results were presented as means and pooled standard errors of the means (**SEM**). Difference was considered significant with *p* < 0.05.

## Results

### Effect of fermented feed on meat ducks growth performance

The effects of FF supplementation on growth performance in meat ducks are illustrated in Table 3. During the early phase, no significant differences were observed in any growth performance parameters between CON and FFD groups during early phrase (*p >* 0.05). During the late phase, ducks in FFD group demonstrated improved growth performance compared with CON. The BW at day 42 (*p =* 0.042) and ADG in late phase (*p =* 0.036) were higher in FFD than CON. Additionally, F/G of ducks in FFD group was enhanced during both the late phase (*p =* 0.032) and entire experimental period (*p =* 0.046). ADFI did not differ between groups at any stage (*p >* 0.050).

**Table 3:** Effect of FF on growth performance of meat ducks.

Item	CON	FFD	SEM	*P*-value
BW (1 d), g	52.24	51.98	2.24	0.603
BW (14 d), g	675.92	705.26	19.92	0.246
BW (42 d), g	3175.00^b^	3266.67^a^	96.26	0.042
ADG (1 to 14 d), g	44.55	46.66	3.81	0.343
ADG (15 to 42 d), g	92.56^b^	94.87^a^	8.24	0.036
ADG (1 to 42 d), g	74.35	76.54	5.95	0.282
ADFI (1 to 14 d), g	64.29	65.71	3.30	0.721
ADFI (15 to 42 d), g	189.63	188.15	15.73	0.902
ADFI (1 to 42 d), g	143.33	142.86	10.66	0.894
F/G (1 to 14 d)	1.44	1.41	0.11	0.437
F/G (15 to 42 d)	2.05^a^	1.98^b^	0.17	0.032
F/G (1 to 42 d)	1.93^a^	1.87^b^	0.13	0.046

FF = fermented feed; BW = body weight; ADG = average daily weight gain; ADFI = average daily feed intake; F/G = feed to gain ratio.

n = 12.

### Effect of fermented feed on meat ducks carcass traits

The effect of FF on the carcass traits of meat ducks is presented in Table 4. The semi-eviscerated yield (*p =* 0.001) and eviscerated yield (*p =* 0.001) were increased compared to the ones in CON. Other carcass traits including weights of carcass, eviscerated carcass, semi-eviscerated carcass, breast muscle, leg muscle, abdominal fat, and sebum, and yields of leg muscle, breast muscle, abdominal fat, and sebum did not differ between two groups (*p >* 0.050).

**Table 4:** Effect of FF on carcass traits of meat ducks.

Item	CON	FFD	SEM	*P*-value
Live weight, g	3276.44	3284.00	91.43	0.491
Carcass weight, g	2855.67	2931.63	84.30	0.356
Semi-eviscerated weight, g	2647.83	2734.59	76.55	0.262
Eviscerated weight, g	2457.33	2694.19	70.72	0.149
Leg muscle weight, g	158.73	171.62	8.30	0.363
Breast muscle weight, g	190.9	215.54	8.18	0.409
Abdominal fat weight, g	22.66	28.17	2.81	0.195
Sebum weight, g	519.53	546.11	35.43	0.752
Semi-eviscerated yield, %	80.80^b^	83.27^a^	0.38	0.001
Eviscerated yield, %	75.00^b^	82.04^a^	0.43	0.001
Leg muscle yield, %	12.96	12.74	0.48	0.579
Breast muscle yield, %	15.57	16.00	0.67	0.662
Abdominal fat yield, %	0.91	1.05	0.10	0.172
Sebum yield, %	21.11	20.27	1.22	0.637

FF = fermented feed.

n = 12.

### Effect of fermented feed on duck breast muscle meat quality and chemical composition

Meat quality parameters and chemical composition of breast muscle are presented in Table 5. Dietary supplementation of FF did not influence the pH_45min_ and pH_24h_, color (45 min and 24 h post-mortem), shear force, cooking loss in the breast muscle between groups (*p >* 0.050). The comparison of chemical composition revealed that crude protein and moisture contents in the breast muscle between groups did not differ (*p >* 0.050). However, crude fat content was lower in breast muscle from FFD group compared to CON (*p =* 0.023).

**Table 5:** Effect of FF on breast muscle quality.

Item	CON	FFD	SEM	*P*-value
pH_45 min_	6.75	6.65	0.13	0.588
pH_24 h_	4.26	4.24	0.01	0.643
L*_45 min_	40.08	41.08	1.47	0.640
a*_45 min_	10.92	10.88	0.46	0.946
b*_45 min_	5.63	5.09	0.43	0.402
L*_24 h_	40.31	41.83	1.21	0.395
a*_24 h_	12.98	11.92	0.63	0.268
b*_24 h_	7.95	7.48	0.63	0.612
Shear force, N	2.06	2.31	0.09	0.179
Cooking loss, %	21.90	21.21	1.71	0.780
Crude protein, %	20.63	20.88	0.29	0.563
Moisture, %	76.92	76.79	0.32	0.785
Crude fat, %	0.82^a^	0.63^b^	0.05	0.023

FF = fermented feed; L* = lightness; a* = redness; b* = yellowness.

n = 12.

### Effect of fermented feed on duck breast muscle amino acid profile

AA composition of breast muscle is presented in Table 6. Among 17 AAs quantified, two AAs showed significant differences between CON and FFD groups. Phenylalanine concentration was higher in FFD group compared with CON (*p =* 0.004). Lysine also was increased in response to dietary FF supplementation and lysine (*p =* 0.038). All other analyzed AAs did not differ between two groups (*p >* 0.050).

**Table 6:** Effect of FF on breast muscle amino acid profile.

Item, %	CON	FFD	SEM	*P*-value
Aspartate	1.75	1.76	0.02	0.598
Threonine	0.87	0.87	0.01	0.577
Serine	0.76	0.76	0.01	0.900
Glutamate	2.87	2.87	0.03	0.968
Glycine	0.88	0.88	0.01	0.788
Alanine	1.15	1.16	0.02	0.753
Valine	0.97	0.99	0.01	0.379
Methionine	0.50	0.50	0.01	0.547
Isoleucine	0.90	0.92	0.10	0.386
Leucine	1.57	1.61	0.02	0.255
Tyrosine	0.68	0.69	0.01	0.173
Phenylalanine	0.79^b^	0.84^a^	0.01	0.004
Lysine	1.63^b^	1.70^a^	0.02	0.038
Histidine	0.53	0.54	0.01	0.341
Arginine	1.24	1.25	0.02	0.552
Proline	0.71	0.73	0.01	0.565

FF = fermented feed.

n = 12.

### Metabolomics of duck breast muscle

**Fig. 1:**
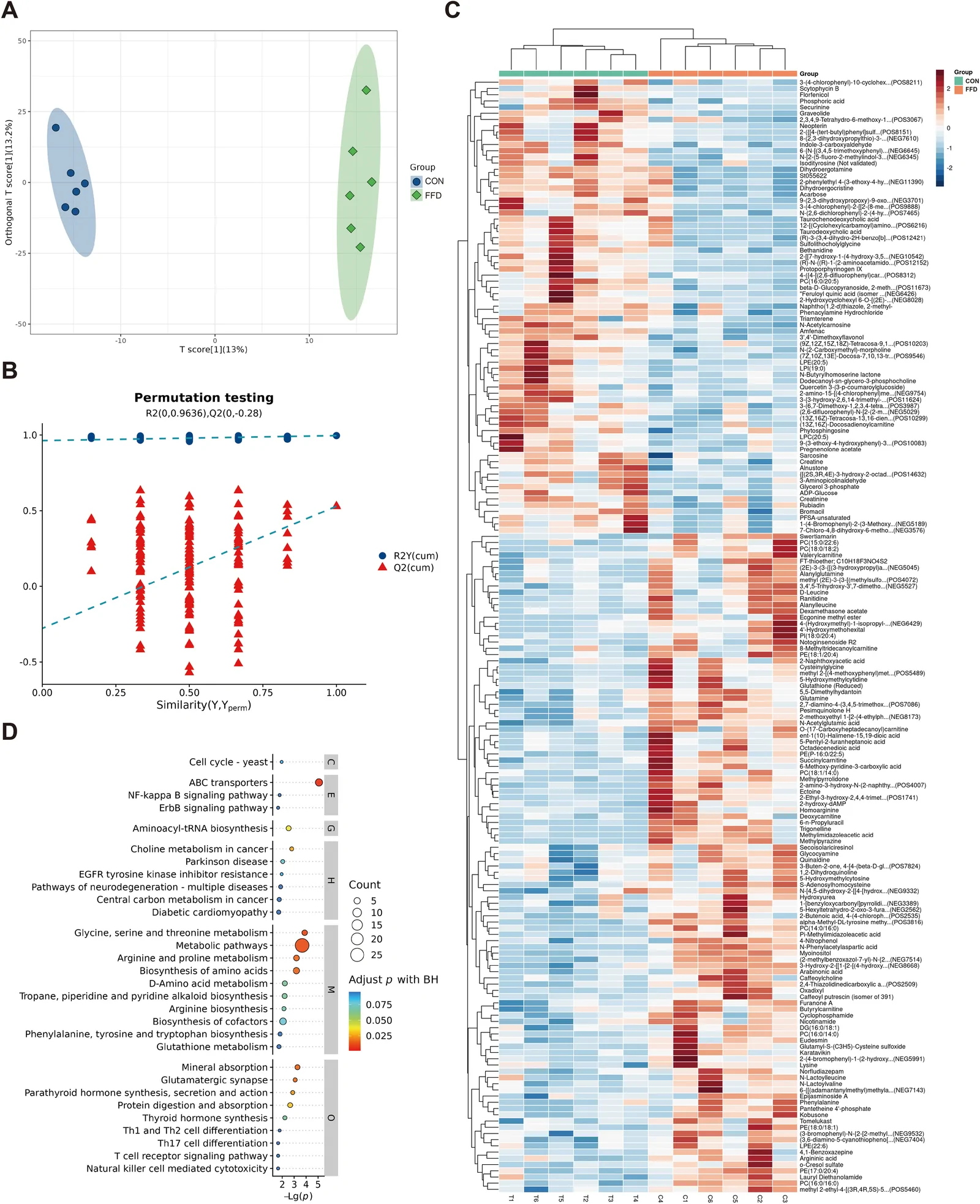
Metabolic analysis of duck breast muscle between CON and FFD groups. (A) Score plot of orthogonal partial least squares discrimination analysis (OPLS-DA). (B) Permutation test on the PLS-DA model. (C) Heatmap and hierarchical clustering of differential metabolites. (D) KEGG Pathway analysis. Each dot represents a pathway and the color indicates the enrichment *P*-value. The size of dot indicates the number of metabolites annotated to this pathway. C, cellular processes; E, environmental information processing; G, genetic information processing; H, human disease; M, metabolism; O, organismal systems.

Score plot of orthogonal partial least squares discrimination analysis (**OPLS-DA**) demonstrated distinct separation in metabolic profiles between breast muscle of CON and FFD groups (Fig. 1A), and permutation test confirmed this pattern was robust for analysis (Fig. 1B). The variable of importance on projection (**VIP**) score derived from OPLS-DA model and *P*-value obtained from Student’s *t*-test were applied to screen out the differential metabolites (**DMs**) under the condition of VI*p >* 1 and *p* < 0.05. A total of 179 DMs were identified, of which 73 DMs were increased in abundance, and 106 were decreased in FFD group relative to CON (Fig. 1C). These 179 DMs mapped to specific biological functions when sorted by KEGG pathway (Fig. 1D), including Cell cycle – yeast in cellular processes, ABC transporters and other two pathways in environmental information processing, aminoacyl-tRNA biosynthesis in genetic information processing, choline metabolism in cancer and 5 more in human disease, glycine, serine and threonine metabolism and 9 more in metabolism, mineral absorption and 8 more in organismal systems.

### Proteomics of duck breast muscle

**Fig. 2:**
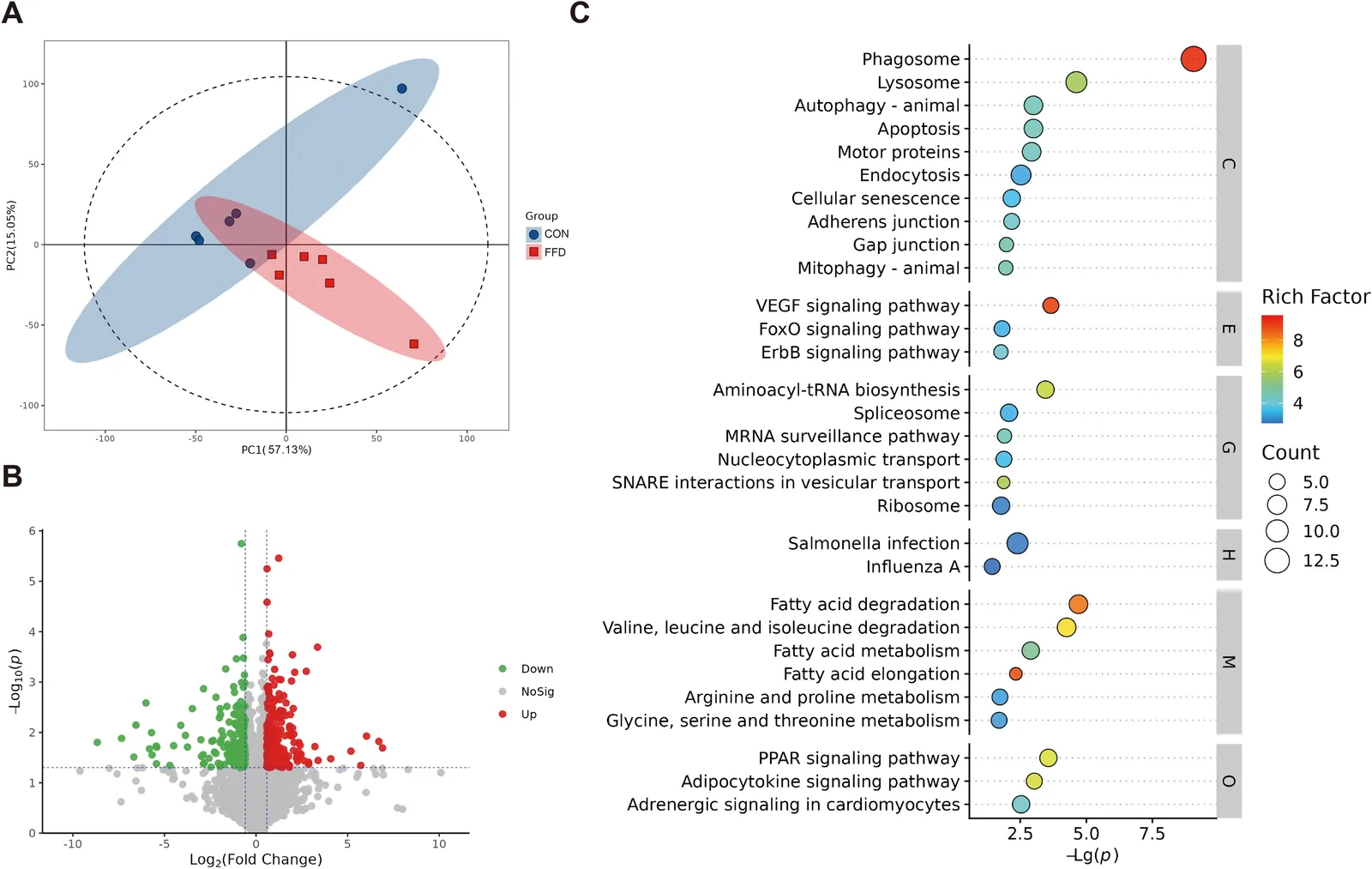
Proteomic analysis of duck breast muscle between CON and FFD groups. (A) Score plot of principal component analysis (PCA). (B) Volcanic map of differentially expressed proteins. (D) KEGG Pathway analysis. E ach dot represents a pathway and the color indicates the enrichment *P*-value. The size of dot indicates the number of metabolites annotated to this pathway. C, cellular processes; E, environmental information processing; G, genetic information processing; H, human disease; M, metabolism; O, organismal systems.

Principal component analysis (**PCA**) illustrated a separation in proteomic profiles in breast muscle between CON and FFD groups, with PC1 and PC2 covering 72.18% of the variability (Fig. 2A). A total of 388 differentially expressed proteins (**DEPs**) were identified with the thresholds of *p <* 0.05 and fold change > 1.5 or < 0.667, of which 220 DEPs were up-regulated, and 168 DEPs were down-regulated in abundance in FFD group compared with CON (Fig. 2B). KEGG analysis categorized these DEPs enriching multiple pathways, including phagosome and 9 more in cellular processes, VEGF signaling pathway and 2 more in environmental information processing, aminoacyl-tRNA biosynthesis and 5 more in genetic information processing, salmonella infection and influenza A in human disease, fatty acid degradation and 5 more in metabolism, and PPAR signaling pathway and 2 more in organismal systems (Fig. 2C).

### Integrated analysis of proteomics and metabolomics of duck breast muscle

**Fig. 3:**
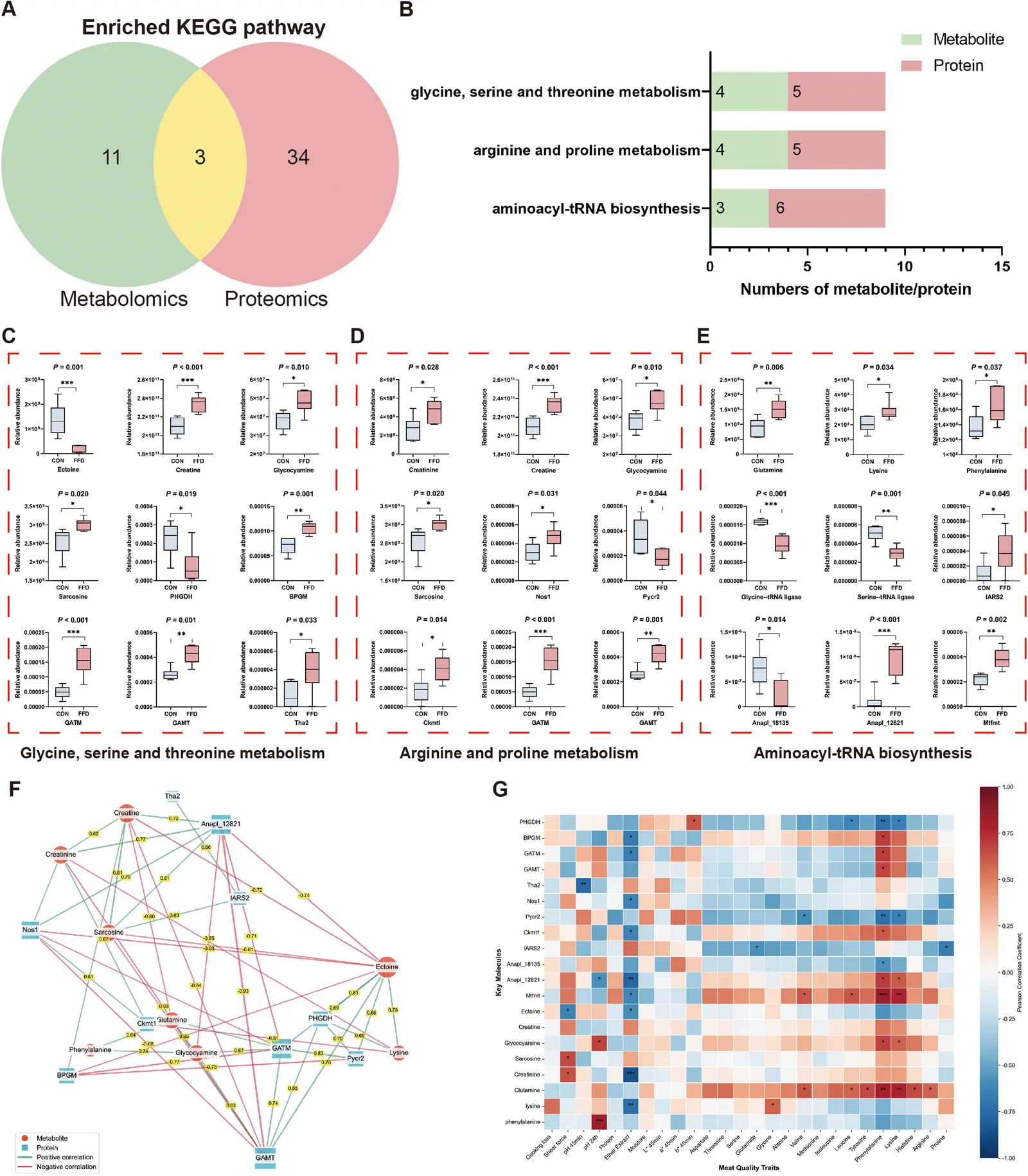
Integrated analysis of proteome and metabolome of breast muscle between CON and FFD groups. (A) Venn diagram of enriched KEGG pathways from metabolomic and proteomic analysis. (B) Numbers of metabolites and proteins involved in the shared KEGG pathways. (C) Changes of metabolites and proteins associating to pathway of glycine, serine and threonine metabolism. (D) Changes of metabolites and proteins associating to pathway of arginine and proline metabolism. (E) Changes of metabolites and proteins associating to pathway of aminoacyl-tRNA biosynthesis. (F) Correlation network analysis of target metabolites and proteins. Nodes represent metabolites (red circles) and proteins (blue squares). Node size is proportional to degree centrality. Edge color indicates correlation direction (green: positive; red: negative). Edge width is proportional to correlation strength. Numbers on edges show correlation coefficients. (G) Correlation analysis between key molecules and meat quality traits (red: positive correlation, blue: negative correlation). *0.01 ≤ *p* < 0.05, **0.001 ≤ *p* < 0.01, ***p < 0.001.

Combining proteomic and metabolomic data uncovered three pathways common to both datasets (Fig. 3A) which potentially contribute to the modulated meat quality influenced by FF. Glycine, serine and threonine metabolism and arginine and proline metabolism contained four DMs and five DEPs that responded to FF. Aminoacyl-tRNA biosynthesis featured three DMs and six DEPs from the two omics (Fig. 3B). In regards to glycine, serine and threonine metabolism, creatine (*p <* 0.001), glycocyamine (*p =* 0.010), and sarcosine (*p =* 0.020) were increased in FFD group, along with enzymes phosphoglycerate mutase (**BPGM**; *p =* 0.001), glycine amidinotransferase (**GATM**; *p <* 0.001), guanidinoacetate N-methyltransferase (**GAMT**; *p =* 0.001), and THA2 aldolase (**Tha2**; *p =* 0.033). But ectoine (*p =* 0.001) and d-3-phosphoglycerate dehydrogenase (**PHGDH**) were down-regulated in FFD (*p =* 0.019; Fig. 3C). In regards to arginine and proline metabolism, creatinine (*p =* 0.028), creatine (*p <* 0.001), glycocyamine (*p =* 0.010), and sarcosine (*p =* 0.020) were increased in FFD group, along with nitric oxide synthase 1 (**Nos1**; *p =* 0.031), creatine kinase U-type, mitochondrial (**Ckmt1**; *p =* 0.014), GATM (*p <* 0.001), and GAMT (*p =* 0.001). But pyrroline-5-carboxylate reductase (**Pycr2**) was decreased in FFD (*p =* 0.044; Fig. 3D). In regards to aminoacyl-tRNA biosynthesis, glutamine (*p =* 0.006), lysine (*p =* 0.034) and phenylalanine (*p =* 0.037) were up-regulated in FFD group, along with isoleucine–tRNA ligase (**IARS2**; *p =* 0.049), leucine–tRNA ligase (**anapl_12821**; *p <* 0.001), and methionyl-tRNA formyltransferase, mitochondrial (**Mtfmt**; *p =* 0.002). But glycine-tRNA ligase (*p <* 0.001), serine‑tRNA ligase (*p =* 0.001), and methionine–tRNA ligase, cytoplasmic (**anapl_18135**) were down-regulated in FFD (*p =* 0.014; Fig. 3E). Pearson correlation analysis of these key molecules identified 24 significant correlations (|r| ≥ 0.6, *p <* 0.050), including 16 positive and 8 negative correlations. A total of 14 out of 18 hub nodes had degree centrality more than 3 (Fig. 3F). Correlation analysis between these key molecules and meat quality traits revealed distinct correlation patterns (Fig. 3G). For example, BPGM, GATM, Nos1, Ckmt1, Anapl_12821, Mtfmt, ectoine, creatinine, and lysine exhibited negative correlations with EE content (*p <* 0.050). BPGM, GATM, GAMT, Ckmt1, Anapl_12821, Mtfmt, glycocyamine, and glutamine showed positive correlations, while Pycr2 and Anapl_18135 showed negative correlations with phenylalanine content (*p <* 0.050). Anapl_12821, Mtfmt, glycocyamine, and glutamine demonstrated positive correlations, while PHGDH and Pycr2 demonstrated negative correlations with lysine content (*p <* 0.050). These findings suggest these key molecules from enriched pathways were closely associated with the modulation of duck meat quality traits in response to FF supplementation.

## Discussion

Fermentation enhances the nutritional value of agricultural by-products through improved palatability and bioavailability, while generating bioactive metabolites that support intestinal health (Zhu et al., 2023). In the present study, dietary inclusion of 5% FF improved growth performance during the late growth phase, but no during the early phase in meat ducks. This phase-specific response likely reflects developmental differences in digestive capacity and nutrient requirements. Early-stage ducklings possess immature digestive systems with limited enzymatic activity and gut microbiota establishment, whereas the late phase coincides with rapid muscle deposition and enhanced digestive competence (King et al., 2000). The FF-derived bioactive compounds, including organic acids, small peptides, and exogenous enzymes, become more bioavailable and functionally relevant in ducks during the late phase when the digestive capacity are fully developed. Moreover, probiotic microorganisms in FF (*Saccharomyces cerevisiae*) modulate gut microbiota composition, enhancing nutrient extraction efficiency during the growth-accelerating late phase (Gao et al., 2022). The observed improvement in feed-to-gain ratio during both the late phase and the entire experimental period confirms that 5% FF inclusion enhances nutrient utilization efficiency in meat ducks. Interestingly, F/G was improved in FFD group with no significant differences in ADFI and ADG for the entire experimental period. This likely reflects the fact that FF primarily influenced growth during the late phase, as evidenced by significant improvements in ADG and F/G during this period. The inclusion of the early phase, when digestive capacity was immature and FF effects were minimal, diluted the overall treatment effects on ADG when calculated across 42 days. However, the consistent improvement in feed efficiency during the lat phase was sufficient to manifest as a significant difference in F/G for the whole experimental period.

Carcass traits represent primary determinants of economic value in meat-type poultry production. In the present study, dietary inclusion of 5% FF improved dressed percentage as well as semi-eviscerated and eviscerated yields, indicating a favorable impact on the carcass traits of meat ducks. These enhanced carcass trats likely result from more efficient nutrient deposition during the late growth phrase, consistent with the improved feed efficiency observed during this period. The combination of better nutrient availability from fermentation and yeast-derived bioactive compounds may support the better slaughter performance (Vlaicu et al., 2025). In line with this result, Zhou et al. (2023) reported that dietary supplement of 3% or 5% FF significantly improved the dressed percentage and semi-eviscerated yield in broiler chicken. Conversely, Soren et al. (2024) detected no significant differences in any carcass traits between broiler chicken consuming a control diet and those receiving *Saccharomyces cerevisiae*-fermented feed additives. The variations among outcomes likely reflects difference in the nutritional composition of FF, as the choice of fermenting substrate and probiotic strain combinations differs substantially across experiments.

Meat quality directly determines economic viability in duck farming. Key parameters include pH, color, texture, cooking loss, and chemical composition, which collectively reflect meat traits such as water-holding capacity, tenderness, juiciness, and nutritional profile, and ultimately influencing consumers’ acceptance (Barbut and Leishman, 2022). In the present study, dietary incorporation of 5% FF reduced CF content in breast muscle without affecting pH values, color, shear force, cooking loss, CP content, or moisture. This pattern aligns with Du et al. (2025), who observed that fermented marigold meal administered at 5% to 10% of dietaries exhibited no significant effects on pH, color, shear force, CP, or moisture within broiler breast muscle. These findings collectively demonstrate FF as a safe and stable feed ingredient for poultry production. Notably, Li et al. (2022) reported that FF inclusion ranging from 6% to 24% of the diet decreased CF deposition in breast muscle of Lande goose. The observed reduction in CF may be attributed to that yeast fermentation produces bioactive compounds including polyphenols and organic acids, inhibiting lipogenesis and stimulate lipolytic pathways (Ahmed et al., 2026). Additionally, *Saccharomyces cerevisiae* possesses lipolytic enzyme activity capable of modulating fat metabolism (Wang et al., 2024). The improved feed efficiency during the late phase may also direct nutrients toward lean tissue deposition rather than fat storage.

AAs constitute the fundamental structural components of muscle tissue. Essential AAs cannot be synthesized by humans and must be obtained from dietary sources (Hou and Wu, 2018). Beyond nutritional value, AAs influence meat flavor through intrinsic taste activity and participation in Maillard reaction and Strecker degradation (Xu et al., 2023a; Indriani et al., 2024). In this study, dietary incorporation of 5% FF significantly elevated phenylalanine and lysine concentrations in duck breast muscle, indicating promotional effects on nutritional quality and flavor potential. These findings align with Hai et al. (2024), who reported that rice bran and maize fermented by *S. cerevisiae* increased phenylalanine and lysine contents in chicken muscle. Comparable outcomes have been documented for fermented oilseed meals, such as peanut, rapeseed, and soybean, where fermentation enhanced the apparent digestibility of phenylalanine and lysine in broilers (Li et al., 2023; Smola et al., 2025; Wu et al., 2020). Given that formulated nutrient levels were equivalent in between CON and FFD, the observed enrichment of specific AAs likely reflects enhanced digestive uptake and absorptive efficiency. It may due to the shifts in intestinal microbiota composition and function induced by fermentation. As proposed by Katu et al. (2025), such microbial modulation can accelerate AA metabolism and transformation, thereby offsetting tissue accumulation for certain AAs while permitting deposition of others. This mechanism elucidates why FF supplementation preferentially enriches phenylalanine and lysine without broadly altering the complete AA profile.

Integrated analysis of non-targeted metabolomics and proteomics identified 179 DMs and 388 DEPs in breast muscle between CON and FF. Pathway enrichment identified glycine, serine and threonine metabolism, arginine and proline metabolism, and aminoacyl-tRNA biosynthesis as shared pathways. The up-regulation of glutamine, lysine, and phenylalanine within these pathways corroborates the targeted AA analysis. These coordinated changes indicate that FF modulates AA metabolic networks in duck breast muscle. In line with this result, Xu et al. (2023b) reported that lactic acid bacteria-derived FF regulated glycine, serine and threonine metabolism in duck meat, and An et al. (2021) observed that fermented *Broussonetia papyrifera* L. activate arginine and proline metabolism in lamb *longissimus dorsi*. Glutamine functions as a key metabolic substrate and structural element within skeletal muscle fibers, and exerts immunomodulatory effects that may contribute to meat quality preservation (Tomaszewska et al., 2025). Lysine and phenylalanine are essential AAs for humans, and their concentrations directly determine the nutritional value of meat products (Hou and Wu, 2018). Although direct microbiome profiling was not conducted, FF likely influenced AA absorption through gut microbiota modulation. Yeast-fermented feed contained prebiotic components including mannan oligosaccharides and β-glucans, which selectively promoted beneficial bacteria such as *Lactobacillus* and *Bifidobacterium* species. These microorganisms enhance intestinal barrier function and upregulate AA transporter expression (Deng et al., 2026). *Saccharomyces cerevisiae*-derived fermentation products including organic acids and small peptides modulated intestinal pH and microbiota composition, creating an environment favorable for proteolytic bacteria that release bioactive peptides from dietary proteins. These peptides could influence muscle AA profiles through the gut-muscle axis (Yang et al., 2025). Collectively, FF exerts significant regulatory influence on the metabolic pathways identified above, inducing favorable alternation in AA and derivative concentrations that contribute to enhanced duck meat quality.

Within these converging pathways from multi-omics analysis, glycocyamine, creatine, creatinine, and sarcosine were upregulated in FF breast muscle, indicating enhanced creatine biosynthesis. Glycocyamine has been reported to enhance meat quality attributes in stressed broilers by elevating pH value while reducing drip loss, cook loss, and L* value (Zhang et al., 2022). Sarcosine modulates meat quality through regulation of water content, muscle volume, water-holding capacity, pH status, and color characteristics (Ma et al., 2020). Creatine functions as a nitrogen-containing organic acid that performs essential roles in ATP regeneration and pH buffering, additionally serving as a bioactive nutrient within meat products (Jia and Zhu, 2023). Creatinine, as a direct derivative of creatine, contributes to energy storage and transfer mechanisms within muscle cells (Kostusiak et al., 2023). In addition, GATM and GAMT, catalyzing creatine biosynthesis from arginine and glycine, were also upregulated by dietary FF. It also warrants consideration for influencing meat quality. GATM is implicated in AA-derived flavor formation processes within chicken breast muscle (Gai et al., 2023). GAMT attenuates stress-induced muscle energy expenditure and quality deterioration in broilers (Zhang et al., 2019). Ckmt1 down-regulation is associated with wooden breast myopathy in broilers (Ding et al., 2026). This coordinated metabolites and their biosynthetic enzymes reflects meaningful enhancements in meat quality, flavor development, and nutrient value, which can be served as candidate biomarkers for FF-induced improvements in duck breast muscle quality.

## Conclusion

In summary, dietary supplementation of 5% FF enhanced growth performance by increasing BW at 42-d-old and ADG for late phase, and decreasing F/G for late phase and whole experimental period. It also improved the carcass traits in meat ducks by improving semi-eviscerated yield and eviscerated yield. Concurrent with these production benefits, FF supplementation reduced crude fat deposition within breast muscle while elevating phenylalanine and lysine concentrations, collectively improving the nutritional quality of duck meat products. Multi-omics analysis further resolved the metabolic architecture underlying these phenotypic improvements, that glycine, serine and threonine metabolism alongside arginine and proline metabolism emerged as central regulatory axes modulated by dietary FF supplementation. Glycocyamine, sarcosine, creatine, creatinine, and their biosynthetic enzymes including GATM, GAMT, and Ckmt1 were identified as key biomarkers reflecting FF-induced improvements in duck breast muscle quality. These findings establish both practical and mechanistic foundation for FF application in duck production system. From an applied perspective, 5% FF inclusion offers a viable nutritional strategy for enhancing duck growth performance, carcass yield, and meat nutritional profile. This work delineated the specific metabolic pathways and molecular signatures through which FF confers its beneficial effects on poultry meat quality, providing mechanistic clarity that can inform future precision feeding approaches and quality control strategies.

## Funding

This work was funded by 10.13039/501100010203China Agriculture Research System of MOF and MARA (CARS-42-21) and 10.13039/501100015335Hunan Poultry Industry Technology System (HARS-06).

## CRediT authorship contribution statement

**Yang Liu:** Writing – review & editing, Writing – original draft, Validation, Methodology, Data curation, Conceptualization. **Hailin Liu:** Validation, Data curation. **Ping Deng:** Visualization, Validation, Software, Data curation. **Chuang Li:** Validation, Resources, Data curation. **Xuan Huang:** Validation, Software, Data curation. **Xu Zhang:** Validation, Formal analysis, Data curation. **Xiaolong Peng:** Validation, Data curation. **Zhicai Li:** Validation, Data curation. **Yan Hu:** Validation, Formal analysis. **Chao Peng:** Data curation. **Qiuzhong Dai:** Supervision, Resources, Project administration, Investigation, Funding acquisition, Conceptualization.

## Disclosures

The authors declare that there is no conflict of interest, and the manuscript is approved by all authors for publication. We would like to declare on behalf of my co-authors that the work described is original research that has not been published previously, and not under consideration for publication elsewhere, in whole or in part. All the authors listed have approved the manuscript that is enclosed.
