# Supplementary material for: Yeast-fermented feed improves growth and meat quality in meat duck: Integrated metabolomics and proteomics reveal amino acid metabolic mechanisms
Source: Poult Sci. 2026 Jun 30;105(11):107366. doi: 10.1016/j.psj.2026.107366 (PMC13589119; doi:10.1016/j.psj.2026.107366)
Supplement: Supplementary file 1 [file mmc1.docx]

**Supplement table 1**. Effect of dietary supplement of yeast-derived FF on growth performance of meat ducks.

| Item | 0 FF | 5% FF | 10% FF | 15% FF | 20% FF | *P*-value |
| --- | --- | --- | --- | --- | --- | --- |
| BW (1 d), g | 52.84±1.52 | 52.82±1.22 | 51.44±1.32 | 53.13±1.83 | 51.71±1.53 | 0.904 |
| BW (14 d), g | 679.11±6.65 | 688.45±8.87 | 690.87±15.77 | 680.41±8.88 | 706.11±12.18 | 0.432 |
| BW (42 d), g | 3185.84±21.26 | 3524.80±45.16 | 3338.38±59.14 | 3203.42±33.33 | 3275.43±26.19 | <0.001 |
| ADG (1 to 14 d), g | 48.17±0.49 | 48.87±0.70 | 49.19±1.25 | 48.25±0.62 | 50.34±0.98 | 0.388 |
| ADG (15 to 42 d), g | 89.53±0.91 | 101.31±1.58 | 94.55±2.08 | 90.11±1.21 | 91.76±1.20 | <0.001 |
| ADG (1 to 42 d), g | 76.42±0.54 | 84.68±1.11 | 80.17±1.43 | 76.84±0.83 | 78.63±0.65 | <0.001 |
| ADFI (1 to 14 d), g | 63.74±1.44 | 64.72±1.49 | 66.53±2.02 | 61.28±1.95 | 63.88±1.54 | 0.314 |
| ADFI (15 to 42 d), g | 192.79±5.23 | 186.94±3.07 | 194.17±4.25 | 183.42±3.61 | 181.52±4.09 | 0.142 |
| ADFI (1 to 42 d), g | 145.68±3.23 | 145.24±3.05 | 140.65±1.97 | 135.11±3.37 | 145.27±2.88 | 0.068 |
| F/G (1 to 14 d) | 1.32±0.03 | 1.33±0.04 | 1.36±0.06 | 1.27±0.05 | 1.27±0.04 | 0.600 |
| F/G (15 to 42 d) | 2.15±0.05 | 1.85±0.02 | 2.06±0.07 | 2.04±0.06 | 1.98±0.05 | 0.004 |
| F/G (1 to 42 d) | 1.91±0.05 | 1.72±0.04 | 1.76±0.05 | 1.76±0.04 | 1.85±0.03 | 0.016 |

FF = fermented feed; BW = body weight; ADG = average daily weight gain; ADFI = average daily feed intake; F/G = feed to gain ratio.

n = 10.
